# Subjective quality of life and sexual functioning after germ-cell tumour therapy

**DOI:** 10.1038/sj.bjc.6601421

**Published:** 2003-12-09

**Authors:** M J Fegg, A Gerl, T C Vollmer, U Gruber, C Jost, S Meiler, W Hiddemann

**Affiliations:** 1 Department of Internal Medicine III, Ludwig-Maximilians-University, Marchioninistrasse 15, 81377 Munich, Germany; 2 Interdisciplinary Palliative Care Unit, Ludwig-Maximilians-University, Marchioninistrasse 15, 81377 Munich, Germany

**Keywords:** quality of life, germ-cell tumour, testicular cancer, sexual functioning, patient-doctor communication

## Abstract

Purpose: To evaluate the influence of germ-cell tumour therapy on sexual functioning and subjective quality of life (QL). To investigate the communication about sexual problems between patients, their partners, and doctors. In all, 474 patients treated for germ-cell tumours at the Department of Internal Medicine III, Ludwig-Maximilians-University Munich, from 1979 to 2000 were asked to complete a self-report questionnaire concerning psychosocial dimensions and subjective QL (QLS; Henrich and Herschbach, 2000). In total, 341 patients returned a completed questionnaire (response rate, 71.9%). The median age at survey was 41.9 years and the median follow-up period after therapy was 9.6 years. Persisting sexual sequelae were lower than in the current literature: decreased sexual desire (7.1%), erection (10.0%), orgasm (10.2%), ejaculation (28.8%), sexual activity (8.5%), and sexual satisfaction (4.8%). In QL the satisfaction with ‘friends/acquaintances’ (*P*<0.001) and ‘family life/children’ (*P*<0.001), is lower than in the healthy population. Correlations between functional scales and subjective QL were highly significant. There is a strong correlation between sexual satisfaction and global life satisfaction (Spearman's Rho: 0.48; *P*<0.01). A total of 61.4% of patients were not offered communication about sexual problems by their doctors and 21.2% were unable to talk with their partner about sexual issues. In conclusion, moderating psychosocial variables (e.g. personality factors, cognitive processes) should be investigated to clarify the relationship between life satisfaction (subjective QL) and functional impairments. Communication about sexual problems should be offered as a standard to patients treated for germ-cell tumours.

Testicular cancer is the most frequent malignancy in young adult males, aged 20–35 years. The incidence has increased significantly over the last two decades (Che *et al*, 2002). Germ-cell tumours are highly sensitive to cisplatinum-based chemotherapy, resulting in cure rates of over 90% for patients with minimal metastatic disease, and in 70–80% of patients with metastatic disease (Bokemeyer *et al*, 1999; Schmoll, 1999; Gerl, 2000). Since most of the patients are going to be long-term survivors with high life expectancy, long-term sequelae (Strumberg *et al*, 2002), side effects and secondary morbidities (Hartmann *et al*, 1999, 2000), as well as psychosocial difficulties, sexual life problems and quality-of-life (QL) issues have become the major long-term complications of testicular cancer treatment. The diagnosis of germ-cell tumours often falls in a life-phase, when young adult men are usually in the prime of their physical health, starting their professional career, and are planning to have a family.

Most studies focus on sexual dysfunction after germ-cell tumour therapy (Bohlen *et al*, 2001; Jonker-Pool *et al*, 2001). Some describe the influence on health-related QL (Fossa *et al*, 1999; Joly *et al*, 2002). Others show the necessarity of an open dialogue between doctors and patients on sexual difficulties (Bourgeois-Law and Lotocki, 1999; Hawighorst-Knapstein *et al*, 2002). Only few studies ask for sexual satisfaction as an area of subjective QL. Subjective QL reflects the difference between an individual's hopes, expectations, and desires and what he or she considers as reality (Calman, 1984).

The WHO defines sexual health as ‘a state of physical, emotional, mental and social well-being related to sexuality; it is not merely the absence of disease, dysfunction or infirmity’. According to this definition, sexual well-being is a complex phenomenon, which comprises physical and psychosocial elements. It also illustrates the difference between sexual functioning and sexual well-being.

There is a broad consensus that two elements are essential to measure QL, namely, multidimensionality and subjectivity (Cella, 1998). Multidimensionality means that the definition must cover different relevant aspects or dimensions of QL, at a minimum physical, mental, and social aspects. Subjectivity reflects the everyday experience that the specific factors determining the quality of a person's life are both highly personal and virtually limitless. Moreover, there are great inter- and intraindividual differences in the perception and evaluation of objective aspects of life or disease.

## 

### Goals of investigation

The present study was designed to evaluate the influences of germ-cell tumours and their therapy on psychosocial dimensions (sexual functioning, social support, communication about sexual problems with partner and doctor) and subjective QL. A specific focus was the relationship between functional parameters and areas of subjective QL.

## PATIENTS AND METHODS

Patients treated for testicular cancer at the department of internal medicine III, Ludwig-Maximilians-University Munich, from 1979 to 2000 were asked to complete a self-report questionnaire concerning psychosocial dimensions and subjective QL. The following eligibility criteria were defined: (1) histologically proven germ-cell tumour or extragonadal germ-cell tumour of the retroperitoneum, (2) absence of progression or second malignancy at survey time, (3) age at least 18 years, (4) absence of psychiatric disease or mental disability.

Clinical staging had been performed according to the TNM classification system. According to the treatment modality, patients were divided into three subgroups: (1) men treated with orchidectomy; (2) men treated with orchidectomy and chemotherapy; and (3) men treated with orchidectomy, chemotherapy, and retroperitoneal lymph node dissection (RLND).

### Questionnaire

As no validated question was available for germ-cell tumour patients in our study, we designed a questionnaire that contained items on general health status, social support, relationship, body image, communication with partner and doctors about sexual problems, and fertility/sexual functioning. The questions in the self-report inventory were of the rating type, mostly ordinal-scaled with five ranks (‘not at all’, ‘slight’, ‘moderate’, ‘quite’, ‘very’).

The questionnaire contained three sections:

1. Sociodemographic data
2. ‘Questionnaire for germ-cell tumour patients in surveillance’ (33 items)
3. ‘Questionnaire on partnership and sexual functioning’ (19 items)

Copies of the questionnaire are available from the authors on request.

**Figure 1:**
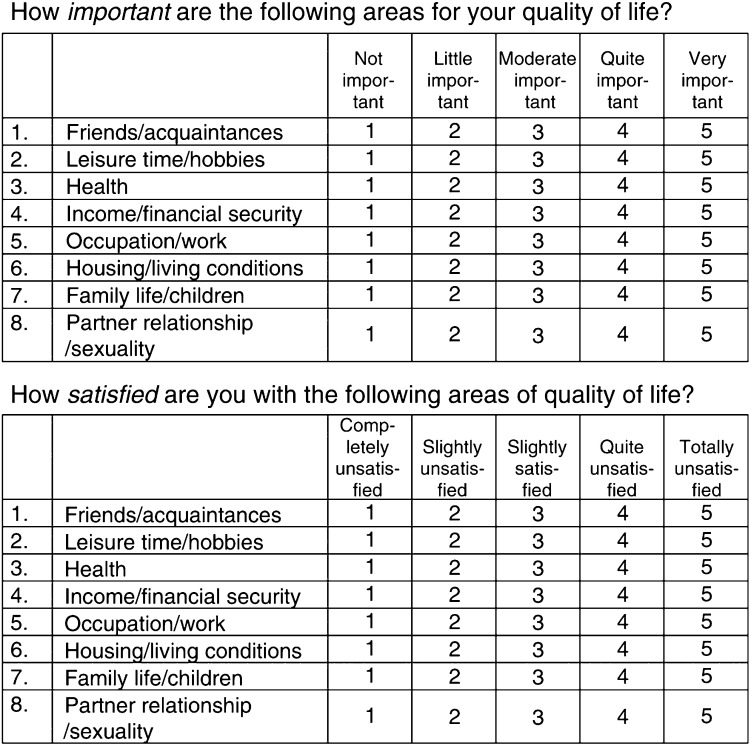
Questions of Life Satisfaction (QLS)

Additionally, the patients received the ‘Questions on Life Satisfaction’ (QLS; Henrich and Herschbach, 2000), which is a validated, short questionnaire for assessing subjective QL (Figure 1

). It covers eight areas of life satisfaction: ‘friends/acquaintances’, ‘leisure time/hobbies’, ‘health’, ‘income/financial security’, ‘occupation/work’, ‘housing/living conditions’, ‘family life/children’, and ‘partner relationship/sexuality’. In the evaluation of the responses, the ratings for importance and satisfaction are combined to yield information about ‘weighted satisfaction’, which varies between −12 and +20. The weighting formula is: wS=importance rating^*^((2^*^satisfaction rating)−3), provided both ratings are made on scales ranging from 0 to 4. The measure of ‘Global Life Satisfaction’ is the sum of the wS values.

### Patients

The questionnaire was sent to 474 patients in April 2000. To include patients who had moved without leaving their new residence address, the residents' registration office was also consulted. Every patient who fulfilled the eligibility criteria was asked to participate. Prior to its initiation, the study was approved by the Ethics Committee of Ludwig-Maximilians-University, Munich.

### Statistical analysis

To portray the clinical relevance of the results in interpretive percentages, the raw scores for the five-point ranking-scales were combined to three categories: ‘not at all/slight’, ‘moderate’, and ‘quite/very’.

The primary results of the study are descriptive, the secondary results inferential. In bivariate analysis (Spearman's correlation), we determined associations between functional limitations and areas of subjective QL. With the Kruskal–Wallis test, we analysed differences for the treatment regimens and the groups after therapy (0–6, 6–12, and over 12 years). *t*-Tests for independent samples were used to investigate the differences between subjective QL in germ-cell tumour patients and healthy Germans. Ordinal regression analysis and MANOVA were used to control the influence of age (at survey, at diagnosis) and time after treatment as confusing parameters on psychosocial consequences and QL.

All *P*-values are Bonferroni corrected. Differences were considered to be statistically significant at *P*⩽0.05. Statistical tests were performed using the SPSS computer program, version 11.0.

## RESULTS

### Participation in the study

From the 474 patients asked to participate, 341 returned a completed questionnaire, 22 refused participation, seven died, and 104 moved untraceably (response rate, 71.9%).

### Respondent characteristics

Table 1

**Table 1:** Patients' characteristics (*n*=341)

**Variable**	**Frequency**	**Percentage**
*Age (years)*		
20–30	38	11.1
30–40	137	40.2
40–50	102	29.9
50–60	44	12.9
60–70	17	5.0
70–80	3	0.9
*Type of disease*		
Seminoma	75	22.0
Non-seminoma	266	78.0
*Stadium of disease*		
1	94	27.6
2	144	42.2
3	103	30.2
*Therapy*		
Orchidectomy	64	19.0
plus chemotherapy	83	24.6
plus RLND^a^	190	56.4
*Family status*		
Single	101	30.8
Partnered/married	203	61.9
Divorced/separated	24	7.3
Widowed	0	0.0
*Partnership (year(s))*		
0–1	27	9.3
1–10	124	42.6
10–20	82	28.2
20–30	35	12.0
Over 30	23	7.9
*Educational level*		
Elementary school	89	26.3
Secondary school	74	21.9
High school degree	67	19.8
University degree	108	32.0
*Employment status*		
Full-time	263	79.2
Part-time	18	5.4
Unemployed	3	0.9
Houseman	2	0.6
In training	16	4.8
Retired	30	9.0

a Retroperitoneal lymph node dissection.

provides an overview of the patients' characteristics. At the time of the study, the median age of the total patient group was 41.9 years (range 22–75 years). At the time of diagnosis, the median age of the total patient group was 31.2 years (range 16–59 years; s.d.=8.7 years). The median follow-up period was 9.6 years after the diagnosis of germ-cell tumour (range 1–22; s.d.=5.7 years). In all, 64 patients (19.0%) underwent orchidectomy and surveillance, 83 patients (24.6%) additional chemotherapy (e.g. PEB, PVB, ECBC), and 190 patients (56.4%) further RLND.

### Sexual functioning and psychosocial sequelae after germ-cell tumour therapy

Table 2

**Table 2:** Sequelae of germ-cell tumour therapy

**Variable**	**Moderate**	**Quite/very**
Decreased general health status	45 (13.2%)	34 (10.0%)
Decreased sexual attractivity	40 (11.8%)	41 (12.0%)
Libido loss	42 (12.4%)	24 (7.1%)
Arousal loss	44 (13.0%)	29 (8.6%)
Decreased erection	36 (10.7%)	34 (10.0%)
Decreased ejaculation	13 (3.9%)	97 (28.8%)
Decreased orgasm intensity	36 (10.7%)	34 (10.1%)

provides information about the reported incidence of post-treatment sequelae after germ-cell cancer therapy (general health status, sexual attractivity, libido, arousal, erection, ejaculation, and orgasm). The decrease in ejaculation depends on therapy regimens (orchidectomy, plus chemotherapy, plus retroperitonal lymph node dissection; Kruskal–Wallis' *χ*^2^: 28.7, df=2; *P*<0.001).

Table 3

**Table 3:** Subjective perceived sequelae through germ-cell tumour therapy – *n* (%)

	**Before therapy**				**After therapy**		
**Variable**	**No/slight**	**Moderate**	**Quite/very**	***P***	**No/slight**	**Moderate**	**Quite/very**
Sexual activity	58 (17.6%)	87 (26.4%)	184 (56.0%)	<0.001	89 (26.9%)	90 (27.2%)	152 (45.9%)
Sexual interest	11 (3.3%)	61 (18.3%)	262 (78.4%)	<0.001	39 (11.7%)	58 (17.4%)	236 (70.9%)
Sexual satisfaction	45 (13.6%)	83 (25.2%)	202 (61.2%)	<0.001	79 (24.0%)	86 (26.1%)	164 (49.9%)

presents information about subjective perceived sequelae through germ-cell tumour therapy. In sexual activity (*P*<0.001), sexual interest (*P*<0.001), as well as in sexual satisfaction (*P*<0.001), there is a highly significant decrease in the patients' perception before and after therapy.

Asked for specific sexual problems before and after therapy, 75 patients (22.0%) reported ‘fear of sexually dissatisfying my partner' after therapy (before therapy: 30 patients, 9.6%; *χ*^2^: 45.6; df=1; *P*<0.001). 32 patients (9.6%) reported ‘inhibitions against my partner’ after therapy (before therapy: eight patients, 2.5%; *χ*^2^: 40.8; df=1; *P*<0.001).

The above sequelae occurred first, in 28 patients (14.4%) ‘immediately after diagnosis’, in 13 patients ‘at the beginning of therapy’, in 27 cases (13.8%) ‘during therapy’, and in 127 cases (65.1%) ‘after therapy’.

There was no significant influence of time between end of therapy and survey point (0–6, 6–12, and over 12 years). Ordinal regression analysis with stage and type of disease as factors, and age (at survey, at diagnosis), as well as years since end of therapy as covariates showed only one significant influence from ‘age at survey’ (positive taxator; *P*=0.001), ‘age at diagnosis’ (negative taxator; *P*=0.005), and ‘time since end of therapy’ (negative taxator; *P*=0.003) on ‘health status after therapy’. This demonstrates that older patients at survey time, younger patients at diagnosis, and patients treated recently for germ-cell tumours report a more severe decrease of health status after therapy. There were no indications in ordinal regression models that younger patients at diagnosis were in advanced stages of their disease. No significant influences were found for the dependent variables ‘sexual attractivity’, ‘sexual arousal’, ‘libido’, ‘quality of orgasm’, as well as ‘sexual activity’, ‘sexual interest’, and ‘sexual satisfaction’.

A total of 117 patients (35.1%) had fathered children before diagnosis (range 1–5; median=2). 196 patients (58.9%) wished to father children after treatment, and 104 patients were unable to do so. In all, 26 patients (25.0%) reported severe distress, caused by the unfulfilled wish for children, 28 (26.9%) reported moderate, and 50 patients (48.1%) ‘no/slight’ distress.

Totally, 293 patients (85.9%) felt strongly socially supported through their family in the process of coping with the disease, 16 patients (4.7%) received moderate, and 32 patients (9.4%) ‘no/slight’ social support.

### Communication about sexual problems

A total of 68 patients (21.2%) were ‘never/seldom’ able to talk with their partner about sexual problems, whereas 188 patients (58.8%) mentioned ‘no/slight’ limitations (64 reported moderate limitations). In all, 35 patients (10.5%) ‘often/always’ wished to have more information on sexual problems from their doctors, 123 (36.9%) ‘sometimes’, and 175 (52.6%) ‘never/seldom’. Totally, 200 patients (61.4%) reported that their doctors did ‘not/seldom’ offer communication about sexual problems, and 75 (23.0%) reported a moderate offer (51 rated ‘often/always’). The wish for more information on sexual problems from the doctors correlates negatively with the quantity of communication between patients and doctors (Spearman's Rho: −0.20; *P*<0.001).

### Subjective quality of life

The results of the QLS in comparison with the healthy German population are listed in Table 4

**Table 4:** *t*-Test on Life Satisfaction on germ-cell tumour patients (*n*=324) and healthy German population (*n*=2534, Henrich and Herschbach, 2000)

**Variable**	**M**	**s.d.**	***P***	**M**	**s.d.**
Friends/acquaintances	6.7	5.9	<0.001	8.1	6.3
Leisure time/hobbies	5.9	5.7	NS	6.3	6.3
Health	8.7	6.6	NS	8.1	7.5
Income/financial security	6.0	5.7	NS	6.5	7.3
Occupation/work	6.0	6.2	NS	5.5	7.3
Housing/living conditions	8.0	5.7	NS	8.3	6.4
Family life/children	8.2	8.2	<0.001	9.8	6.9
Partner relationship/sexuality	7.3	8.7	NS	7.9	7.7
Global Life Satisfaction	56.7	30.8	NS	60.5	37.3

. There are highly significant differences in the dimension ‘friends/acquaintances’ and ‘family life/children’. Patients treated for malignant germ-cell tumours are less satisfied with these areas.

A MANOVA showed no significant influences of the factors ‘stage’ and ‘type of disease’ and of the covariates ‘age’ (at survey, at diagnosis) and ‘years since end of therapy’ on the areas of life satisfaction.

Sequelae in ‘general health status’, ‘orgasm intensity’, ‘partner communication on sexual problems’, ‘sexual activity’, and ‘sexual satisfaction’ are correlated highly significantly with several areas of life satisfaction. Table 5

**Table 5:** Correlation between sequelae of germ-cell tumour therapy and Life Satisfaction (Spearman's Rho ⩾0.30; *P*<0.01)

	**Areas of Life Satisfaction**				
**Sequelae in …**	**Health**	**Occupation/work**	**Family life/children**	**Partner relationship/sexuality**	**Global life satisfaction**
General health status	0.42	0.30	NS	NS	0.30
Orgasm intensity	NS	NS	NS	0.30	0.32
Partner-communication on sexual problems	NS	NS	0.30	0.33	0.39
Sexual activity	NS	NS	NS	0.35	NS
Sexual satisfaction	0.31	NS	NS	0.62	0.48

illustrates highly significant correlations with a Spearman's Rho ⩾0.30.

## DISCUSSION

The present study demonstrates several correlations between sexual functioning and subjective QL, assessed by the QLS. In contrast to other QL measures, the QLS comprises levels and weights of life satisfaction. Our data suggest that there are long-lasting differences between the QL of germ-cell tumour patients and healthy Germans. Patients seem to have less satisfactory social contacts with their ‘friends/acquaintances’. This might come from long-lasting social avoidance, or impeding inhibitions of patients to talk openly about their disease (Flanagan and Holmes, 2000). Interestingly, no significant correlations were found between the collected data and this area.

The unfulfilled wish for children in 104 patients may explain the lower values of satisfaction in ‘familiy life/children’, as 51.9% reported moderate or severe distress. Childlessness is often a major threat to QL in testicular cancer survivors (Rieker *et al*, 1990). The number of patients who had already fathered children before diagnoses (35.1%) parallel the results from an earlier study (Bohlen *et al*, 2001). Additionally, limited partner communication about sexual problems is correlated with this area; 41.2% of patients mentioned moderate-to-severe limitations in their partner dialogue.

Although there were several limitations in sexual functioning, the life satisfaction in ‘partner relationship/sexuality’ did not differ significantly from the healthy German population. Most correlations are weak, supposing other, modulating psychosocial variables (e.g. personality, cognitive processes).

The prevalence rate of sexual dysfunction in this paper is lower than that in the literature. Jonker-Pool *et al* (2001) reviewed publications from 1975 to 1999 in a meta-analysis (36 studies with a total of 2786 cases). In the present study, the patients reported lower limitations in sexual desire (7.1 *vs* 19.6%), erection (10.0 *vs* 11.5%), orgasm (10.2 *vs* 19.8%), ejaculation (28.8 *vs* 44.2%), sexual activity (8.5 *vs* 24.2%), and sexual satisfaction (4.8 *vs* 19.1%); they are older (41.9 *vs* 34.7 years), and the follow-up is more extended (9.6 *vs* 6.9 years). The recent study of Incrocci *et al* (2002) also reports higher functional problems. Since most of the studies in this research area used self-designed questionnaires, it is difficult to compare their results. Standard instruments, containing normative data, should be developed for investigation in sexual sequelae of cancer patients.

The younger the patients at diagnosis, the shorter the time between treatment and survey, and the older the patients at survey time, the higher were the impairments in general health status. Since the last factor covers the ageing process, and the medium factor depends on persisting treatment side effects, the first factor could describe worsening effects of cancer treatment on general health status, especially in young adult males (Greenberg *et al*, 1997; Bauld *et al*, 1998).

Not surprisingly, the ejaculatory dysfunction depended on treatment regimens.

The treatment of germ-cell tumours seems to have highly significant influences on sexual activity, interest, and satisfaction. Asked when sexual problems first occurred, 21.1% reported ‘immediately after diagnosis’ or at the ‘beginning of therapy’. This indicates that sexual dysfunction in testicular cancer also implies psychological factors (van Basten *et al*, 1997), besides somatic side effects of therapy.

There are disillusioning results on the patient–doctor communication about sexual problems. As 21.2% of patients ‘never/seldom’ were able to talk with their partner about sexual problems, 61.4% reported that their doctor, treating for germ-cell tumour, did ‘not/seldom’ offer communication about sexual problems. This needs further investigation, to also assess the doctors’ rating and eliminating possible recall biases. In the total German population, only 11% of the patients were asked by their doctor about sexual problems in the last 3 years (Hartmann *et al*, 2002).

In conclusion, the present study shows lower limitations in sexual functioning than previously reported in the literature for germ-cell tumour patients. There seem to be persisting impairments in life satisfaction in ‘friends/acquaintances’ and ‘family life/children’. The correlations between subjective QL and functioning scales are highly significant. There is a strong correlation between sexual satisfaction and global life satisfaction. Modulating psychological variables (e.g. personality, cognitive processes) should be investigated. Sexual problems should be discussed with all patients before the start of treatment and at follow-up examination (Kunkel *et al*, 2000).
